# Local Anæsthesia in General Surgery

**Published:** 1906-06-16

**Authors:** 


### Local Anæsthesia in General Surgery.

The subject of local anæsthesia in general surgery has not attracted much attention in England, although a few surgeons have advocated its advantages very strongly. The reason is not far to seek, at any rate in London, where there exists a group of trained men who are skilful anæsthetists, and devote their entire time to the administration of anæsthetics. In the Latin countries it has not hitherto been found possible to obtain the services of special anæsthetists, and the value of the skilled production of insensibility during a surgical operation is not yet sufficiently appreciated by the public to make it possible for medical men to specialise in this branch. It happens, therefore, that in Great Britain most of the junior members of the profession have been taught the method of producing general anæsthesia, and are not afraid of administering gas, ether, and chloroform, whilst in France, in Spain, in Portugal, and in Italy local anæsthesia has come into very considerable use.

Dr. Struthers, of Edinburgh, has recently published a very valuable, interesting, and well written book entitled "Notes on Local Anæsthesia in General Surgery," showing that we have erred in adhering too exclusively to general anæsthesia in many cases where shock, discomfort, and even danger might have been avoided by resorting to cocaine, eucaine, or stovaine. Opinions differ widely, as he points out, concerning the extent to which local anæsthesia may be employed legitimately in the interests of the patient. Some require a general anæsthetic even for the most trivial operations; others prefer local anæsthesia, for they dread the chloroform more than the operation. Speaking generally, it may be said that the operations, which may be done suitably under local anæsthesia, are those in which the field of operation is comparatively limited and superficial, and in which its limits can be outlined with tolerable certainty before the operation is begun. Operations in deeply seated tissues, on tumours, etc., of uncertain extent and wide attachments are unsuitable for local anæsthesia, and if it is used in such cases a not uncommon result is the infliction of severe pain on the patient or the performance of an incomplete, and therefore bad, operation. In estimating the possibilities attending the use of local anæsthesia, too, it must be borne in mind that the sensibility of the tissues in the body varies greatly. The subcutaneous fat, muscles, tendons, and fasciæ are very much less sensitive to pain than the skin, and are in fact almost insensitive. Periosteum is highly sensitive, but bone denuded of periosteum is insensitive, so that ribs bared of periosteum may be painlessly excised in operating for empyema, and exostoses may be chiselled off when the periosteum has been rendered analgesic by a cocaine-adrenaline solution.

The methods of producing local anæsthesia, whether by infiltration, by regional, or by spinal anæsthesia, are by no means perfect. They are attended by some grave disadvantages, as is very frankly pointed out by Dr. Struthers, and they have some dangers peculiar to themselves. They are only applicable to certain cases, but Dr. Struthers makes out a good case of their use, and gives in detail the precautions which must be taken to obtain the best results.

